# How nursing students’ placement preferences and perceptions of community care develop in a more ‘community-oriented’ curriculum: a longitudinal cohort study

**DOI:** 10.1186/s12912-020-00473-3

**Published:** 2020-08-26

**Authors:** Margriet van Iersel, Corine H. M. Latour, Marjon van Rijn, Rien de Vos, Paul A. Kirschner, Wilma J. M. Scholte op Reimer

**Affiliations:** 1 Centre of Expertise Urban Vitality, Faculty of Health, Amsterdam University of Applied Sciences, Tafelbergweg 51, 1105 BD Amsterdam, the Netherlands; 2 Department of Internal Medicine, Section of Geriatric Medicine, Amsterdam University Medical Center, Amsterdam, the Netherlands; 3 Centre of Evidence Based Education, Amsterdam University Medical Center, Meibergdreef 9, 1105 AZ Amsterdam, the Netherlands; 4 Open University of the Netherlands, Heerlen, the Netherlands; 5 Open University of the Netherlands / ExCEL, Thomas More University of Applied Sciences, Valkenburgerweg 177, 6419 AT Heerlen, the Netherlands; 6 Department of Cardiology, Amsterdam University Medical Center, Amsterdam, the Netherlands

**Keywords:** Career choice, Community care, Curriculum design, Nurse education, Perceptions, Placement preferences

## Abstract

**Background:**

Extramuralisation in healthcare has influenced medical and nursing curricula internationally with the incorporation of themes related to primary/ community care. Despite this, students do not easily change their career preferences. The hospital is still favourite, leading to labour market shortages in extramural care. This study investigates how baccalaureate nursing students’ perceptions of community care and placement preferences develop over time in a more ‘community-care-oriented’ curriculum, to gain insights on which curriculum elements potentially influence career choices.

**Methods:**

A nursing student cohort of a University of Applied Sciences in the Netherlands (*n* = 273) underwent a new four-year curriculum containing extended elements of community care. The primary outcome was assessed with the Scale on Community Care Perceptions (SCOPE). Data were collected each year of study. Descriptive statistics were used to investigate students’ placement preferences and perceptions, and linear mixed model techniques (LMMs) for measuring how students’ perceptions develop over time. Patterns of placement preferences at individual level were visualised.

**Results:**

Students’ perceptions of community care, as measured with SCOPE, show a slight decrease between year 1 and 4, while items mutually differ substantially. In contrast, the preference of community care for a placement increases from 2.6% in year 1 tot 8.2% in year 4. The hospital is favourite in year 1 (79.8%), and remains most popular. At individual level, students often change placement preferences, although a preference for the hospital is more consistent. The LMMs indicates that, at the four time-points, the estimated marginal means of students’ perceptions fluctuate between 6 and 7 (range 1–10). A placement in community care did not positively influence students’ perceptions, and an intensive 1 week theoretical programme was only temporarily influential.

**Conclusions:**

Although interest for placement in community care increased substantially, it was not clear which curriculum elements stimulated this, nor did the curriculum positively influence students’ perceptions. As most students do not look forward to the high responsibility of the field, other curricula with educational tracks for more mature students/ nurses with a vocational training may be an alternative contribution to solving the labour market problems in community care.

## Background

The extramuralisation of healthcare has influenced the content of baccalaureate nursing education in many Western countries. Nursing educators have redesigned the curricula in line with new educational profiles based on this healthcare development, incorporating themes related to caregiving in the community [1, 2], also in the Netherlands [3]. However, despite this increased extramural orientation in nursing education, many nursing students still orientate on the hospital for their future employment, an environment they see as most attractive [4–8]. Students’ career choices contribute to widespread labour market shortages in community care [9, 10]. This is not unique for nursing education, a similar process is taking place in medical education with increasing deficits in primary care [11–14].

The discrepancy between labour market demands and students’ career choices raises the question whether institutions in healthcare education can help solving this societal problem. As there is a direct association between perceptions of healthcare areas and career choice [15, 16], integrating curriculum elements that stimulate specific career choices by influencing students’ perceptions of less popular areas could be worth a try. Such an approach seems promising since most research reveals that students’ perceptions of healthcare areas, and thus their future plans, tend to shift while they progress through their undergraduate programme, in nursing [4, 8, 16] and medical [15, 16] education. Therefore, insight into curriculum elements that potentially stimulate positive interests in community/ primary care is necessary.

Placement experiences have proved to be influential, as exposure to the professional practice appeared to positively influence students’ perceptions in community nursing [5, 10, 17–22] and in primary care [13, 23]. Educational nursing programs on elderly care have proved to be successful in stimulating students’ interest [24–27], and a simulated short community event recently successfully promoted the relevance and importance of community nursing to students in the UK [28]. Despite these short-term positive results, a literature study by Pfarrwaller et al. revealed that, in the medical context, isolated modules were not effective in stimulating a choice for primary care, which led to the advice to develop a longitudinal, multifaceted programme [12]. However, this type of approach in a nursing curriculum to positively influence students’ perceptions of community care, performed by the authors of the present study, showed no significant effect [29]. As in this former study the effect of the curriculum as a whole was measured, a subsequent longitudinal study is required to further examine how nursing students’ perceptions of community care and placement preferences develop over time in a more ‘community-care-oriented’ curriculum.

### Aim of this study

The general objective of this study is to formulate recommendations for a baccalaureate nursing curriculum stimulating a positive interest in community care, based on knowledge on how students’ perceptions of community care and placement preferences develop while progressing through the curriculum.

The research questions are:

1. How do baccalaureate nursing students’ perceptions towards community care and placement preferences develop during a more community care-oriented curriculum?
2. How do students’ perceptions interact with specific curriculum elements, i.e., which elements are potentially influential in achieving a more positive perception of community care?

## Methods

### Design

A longitudinal cohort survey study was carried out in a single institution. Nursing students underwent a redesigned curriculum with extensive elements of community care.

### A model for influencing perceptions

To influence students’ perceptions, a psychological model was chosen, describing how individuals respond to stimuli (here, the curriculum-redesign), namely *affective* and/or *cognitive*. Affective responses are fluctuating positive or negative emotions varying in intensity, and cognitive responses are ideas about how attractive a specific object (e.g., community care) is seen to be [30]. This perspective of affective and cognitive responses fits in well with the DAGMAR model - Defining Advertising Goals for Measured Advertising Results, a model for marketing and communication [31]. This model explains how individuals perform in decisions about purchases in four steps, which, in this example, is a process that begins with (1) the awareness about the possibility of a career in community care, moves to (2) an understanding what the career brings with it, then (3) to the belief about the career being the right choice, and (4) ends with a practical action: making the career choice. During this process, people progress through three successive phases: *cognitive* (thinking), *affective* (feeling), and *conative* (doing). Therefore, the approach of implementing a more community-care-oriented curriculum implies that students will have increased knowledge about this area (cognitive), possibly leading to a growing appeal, due to a sense of the field’s attractive aspects (affective), and subsequently a choice for a future career in this area (conative).

### Participants and method of data collection

Nursing students from one University of Applied Sciences in a larger city in the Netherlands took part in the study. Longitudinal data collection took place four times from a single cohort of students in the full-time programme between October 2014 and May 2018 (the duration of the Dutch bachelor of science nursing programme is 4 years). Students in other educational pathways and/or specific programmes, and students that underwent only a part of the intervention due to enrolment in year two were excluded. The students were approached during allocated class time and, if not present, individually by email, in order to increase response rate. Data on students’ perceptions and placement preferences were collected with a questionnaire (see below).

### Ethical considerations

The Ethical Review Board of the Open University of The Netherlands approved the study (reference U2014/07279/HVM). Students received information about the research project via the digital learning environment of the university. A letter was published about the purpose of the project, highlighting ethical aspects such as confidentiality of the information, and who would have access to the data. It was also emphasised that non-participation would in no way impact their studies. This was repeated during the data collection in the student groups, and verbal informed consent was given by all respondents.

### Instrument

For the survey, the Scale on Community Care Perceptions (SCOPE), a valid and reliable instrument (Cronbach’s α = .892), was used [32, 33]. SCOPE measures students’ perceptions of community care, placement preferences and underlying assumptions in 35 items. The first part of the instrument consists of items on potentially influential demographics. Students’ perceptions are measured in 33 items in three subscales: (1) the affective component of community care perception, (2) perception of a placement in community care, and (3) of community nursing as a profession. Items range from 1 (negative adjective) to 10 (positive adjective). The placement and profession subscales are aspects of cognitive components of students’ perceptions of community care. These two scales contain the extra option ‘I don’t know’, as it is relevant to gain an insight on aspects that students think they lack knowledge.

Finally, the student chooses a preferred area for a current placement from six healthcare areas: medical rehabilitation, mental healthcare, care for mentally disabled, community care, elderly care, and the general hospital. Then three aspects from the earlier profession scale that primarily determine the preference for the chosen area are selected. As this questionnaire measures the affective and cognitive component of perceptions [34], it is well aligned with the aforementioned DAGMAR-model.

### The intervention: curriculum-redesign

The renewed curriculum, designed with the purpose to stimulate a positive interest in community care, contained an approach based on three elements: (1) influence of lecturers, (2) students’ experiences in the practice of the profession during placements and (3) new theoretical themes in the in-school curriculum.

With regard to the first element, the intervention focused on communication of lecturers to students about different areas. In a workshop to prepare curriculum redesign, reflecting on their own perceptions, many lecturers noticed they implicitly or explicitly advocated their own professional history (often related to hospital care) as a reference point. Therefore, new lecturers were recruited with a lot of expertise and/or experience in community nursing, acting as role models. Guest-lectures performed by community nurses were organised with challenging patient cases from the daily professional practice.

Second, management representatives from school and community-care organisations collaborated to ensure that a placement in the field was considered a positive experience for students. Mentors with a suitable level of education, i.e., a bachelor’s degree, are crucial in ensuring that students meet their learning needs [35–37]. However, as a result of labour market shortages, good mentors are not available everywhere.

Third, a new in-school curriculum was implemented. To understand the structure of this curriculum-redesign, an image, by and large, of the 4-year curriculum, is needed. Year 1 and 2 include general/broad theory about all types of patients in different contexts. In year 2, all students choose a one semester/ 20 week minor-programme (30 EC) for year 3, based on a specific theme or healthcare area (e.g., health technology, mental health, community care). The other 20 weeks in year 3, and the first 30 weeks in year 4 contain two different placements, with the placement in year 4 in a, by the student, preferred healthcare area. As a consequence, students have the opportunity to create an individual pathway in their study in the last 2 years of their study, based on their own interests and mostly relating directly to their career preferences.

**Fig. 1:**
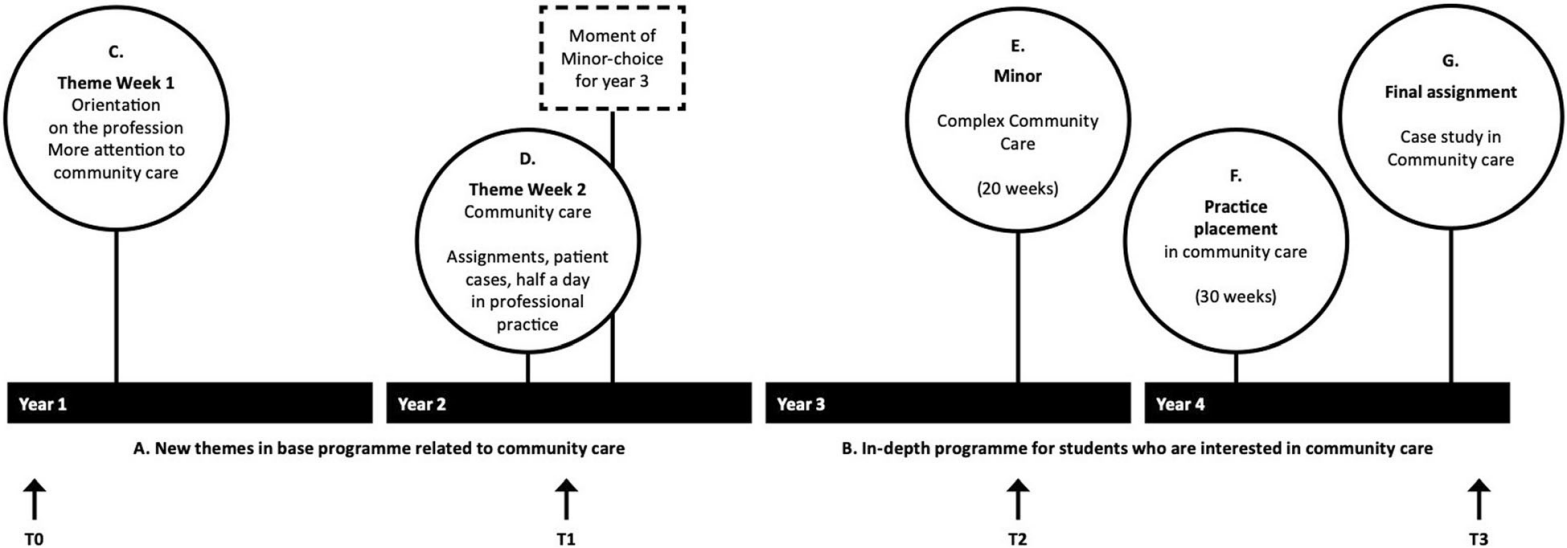
Curriculum redesign stimulating a positive interest in community care, and planning of data-collection (T0–3)

The first purpose of the new programme (see A in Fig. 1) was to broaden students views on the nursing profession, convincing them that nursing is more than hospital care. To get a sense of how/ which patient cases in the lessons were presented, all course materials were analysed. It appeared that, although many of these cases did not refer to a specific healthcare area, more than 60 of 110 cases took place in the hospital, compared to only four in community care. This imbalance, being an aspect of the ‘hidden curriculum’, was corrected by adding more patient cases in community care. By this time, a new more ‘community-oriented’ national Dutch profile for bachelor nursing education was developed [3]. Five themes from this profile were integrated in the broad theory programme of the curriculum: (1) fostering patient self-management, (2) shared decision-making, (3) collaboration with the patients’ social system, (4) healthcare technology, and (5) allocation of care.

In year 1 and 2 (period A in Fig. 1), the specific elements in the new curriculum were:

- An introduction week at the start of year 1, presenting a broad picture of the profession with extra attention for community nursing (C, Fig. 1).
- A ‘Community Care Week’ in year 2, intentionally planned shortly before students’ minor-choice for year 3. All students visited a nursing team working in people’s own homes. Where possible, they participated in the caregiving. The week further consists of assignments on analysing patient cases, a digital game, and ‘speed dates’ showing the diversity of community nursing, with nurses from palliative care, a technical team, and children home care (D, Fig. 1).

The second purpose of the curriculum redesign was to create a ‘paved way’ in the direction of a future career in community care, by offering a challenging in-depth programme in year 3 and 4 (period B in Fig. 1):

- A minor ‘Complex Community Care’, with theory on challenging topics on community nursing: population-based prevention, multimorbidity, interprofessional collaboration, professional leadership, and system-based communication (E, Fig. 1).
- A practice placement in the fourth year, where the student works on all competencies required for the independent role of the community nurse. During this placement, a complex patient case is selected by the student for analysing the caregiving in the final assignment (F, Fig. 1).
- A graduation paper, being a case study of the selected patient. In short, the student uses clinical reasoning in analysing health problems and chooses substantiated evidence-based interventions for patient care (G, Fig. 1).

### Planning of data collection

Four moments of data collection between 2014 and 2018 (T0, T1, T2 and T3, Fig. 1) were carefully planned to measure the effect of specific interventions. T0 and T3 were planned as close as possible to the beginning and the end of the educational programme. T1 was planned shortly after the community care week in year 2 (intervention D), and T2 at three-quarters of the third year when all third-year students by that time had experienced a placement and (at least a part of) the minor-programme of their choice.

### Data analysis

Descriptive statistics were used to summarise student characteristics. The five negatively formulated items in the affective component scale of the SCOPE scale were recoded. Dimension scores were calculated by adding up all items and dividing them by the number of items, in the total scale and subscales, in order to give equal weight to each scale [38]. The normality of the data distribution was assessed, showing that assumptions for using parametric statistics were fulfilled. The student information system (SIS) was used for data on students’ factual placements. Students’ placements preferences at cohort level from T0 to T3 (percentage, n) were analysed using contingency tables. To examine whether and how students’ placement preferences for community care and the hospital fluctuate during their study, students’ preferences were analysed at the individual level and visualised in schemes (see Appendix 1 for the visualisation schemes of placement preferences with regard to the other four healthcare areas).

To analyse students’ perceptions of community care, first, descriptive statistics (mean, SD) were used at the four time points at the level of total scale, subscale and item. Second, the development of students’ perceptions, i.e., the effect of time (T0-T3) on perceptions, was analysed using linear mixed model techniques (LMMs). This technique is specifically suitable for analysing a repeatedly measured continuous dependant variable (perceptions/ total scale), when (1) the observations over time are not independent of each other and (2) missing data occur from students not participating at all four time points due to drop out [38–40]. The data were analysed using IBM SPSS® version 25 (IBM Corporation, Armonk, NY).

## Results

### Participants and response rate

In total 281 students started in the cohort. The size of the sample decreased 35% from 281 (T0) to 183 (T3) due to drop-out, study delay for longer than one semester, and a choice for another educational pathway during study. The response rate was high: in year 1 (T0), 273 of 281 students (97%) responded, in year two (T1), 188 of 206 students (91%), in year three (T2), 166 of 184 (90%), and in year four (T3), 170 of 183 (93%). The number of students of which data were collected at all four time points was 135.

### Demographics

Of the 273 first-year students who filled in the questionnaire at T0, 239 (87.5%) were female and 34 (12.4%) male. Mean age was 19.8 years (SD = 2.7, range 16–44). Of the 273, 225 (82.4%) had completed general secondary education, 43 (15.8%) had completed secondary vocational education, and 5 (1.8%) had completed higher education. The number of students born in the Netherlands was 252 (92,3%). Many students had, in some way, experience with community care: 44.0% had family or friends working in the field, 18.3% (had) worked themselves in some form of community care, and 35.2% had, either themselves or a close family member, received home care (note: an overlap in positive response to these three items is possible). The students’ demographics correspond approximately with a larger sample (*n* = 1058) in a multicentre study in the Netherlands [6] and, therefore, can be considered to be typical for Dutch nursing bachelor programmes. However, as this University of Applied Sciences is located in the multicultural environment of a large Dutch city, the number of students born in the Netherlands in the present sample is somewhat lower (92.3% versus larger sample 95.8%).

### Placement preferences at cohort level

On the question: “If you were to begin your practice placement next week, where would you choose to do it?”, the majority of the students chose a general hospital in year 1 (79.8%), and this remains the most popular during the 4 years of study. In the development of the placement preferences, no significant differences between male and female students were observed. Mental healthcare is the second popular area at T0 (12.8%), decreasing to 8.8% at T3. Community care is the least popular area at T0 (2.6%), fluctuates during the four time points but increases between T0 and T3, to almost the same level as mental healthcare at T3 (8.2%). This changed the rank order of preference for community care from 6 at T0 to 3 at T3 (Table 1).

**Table 1:** Description of students’ placement preferences at T0–3

	T0 (*n* = 273) % (*n*)^a^	T1 (*n* = 188) % (*n*)^a^	T2 (*n* = 166) % (*n*)^a^	T3 (*n* = 170) % (*n*)^a^
General hospital	79.9 (218)	75.0 (141)	78.9 (131)	76.5 (130)
Mental healthcare	12.8 (35)	8.0 (15)	11.4 (19)	8.8 (15)
Medical rehabilitation	4.8 (13)	3.7 (7)	3.0 (5)	3.5 (6)
Care for mentally disabled	3.3 (9)	4.8 (9)	1.8 (3)	2.9 (5)
Elderly Care	2.9 (8)	1.1 (2)	.06 (1)	.06 (1)
Community Care	2.6 (7)	8.5 (16)	3.6 (6)	8.2 (14)

^a^Some students mentioned more than one option, which caused a total score at T0, T1 and T3 of > 100%. Based on the assumption that they did not read the instructions well, and preferred more than one field, this information was not excluded

### Perceptions of community care at cohort level

The description of students’ perceptions of community care at T0-T3 (SCOPE, total scale and subscales, range 1–10 (Table 2)) shows a slight increase of the mean in the total scale and the three subscales between T0 and T1, but an overall small decline between T0 and T3 (total scale 6.66 vs 6.21, respectively), with the lowest score and the largest decline in the placement scale (6.23 vs 5.51, resp.).

**Table 2:** Description of students’ perceptions of community care at T0-T3 (SCOPE: total scale, subscales, and per item)

Perceptions: range 1–10 in mean (SD) with mean values < 5.5 and > 8 in bold	T0 (*n* = 273)	Missing at T0	T1 (*n* = 188)	Missing at T1	T2 (*n* = 166)	Missing at T2	T3 (*n* = 170)	Missing at T3
SCOPE: total scale (33 items)	6.66 (0.94)	0	6.68 (1.07)	1	6.14 (1.12)	0	6.21 (1.08)	0
Affective component scale (11 items)	6.64 (1.21)	2	6.72 (1.24)	1	6.44 (1.40)	0	6.55 (1.25)	0
Placement scale (5 items)	6.23 (1.54)	7	6.37 (1.50)	5	**5.41** (1.64)	0	5.51 (1.58)	3
Profession scale (17 items)	6.80 (0.96)	1	6.98 (0.99)	2	6.56 (0.93)	0	6.57 (0.92)	0
Affective component scale								
Dull - interesting	5.86 (2.18)	3	6.21 (2.04)	1	5.82 (2.08)	0	5.81 (2.17)	0
Boring – fascinating	6.24 (2.04)	6	6.13 (2.09)	2	5.65 (2.10)	0	5.75 (2.11)	1
Unpleasant – pleasant	6.18 (1.79)	5	6.25 (1.73)	3	5.95 (2.04)	1	6.07 (1.98)	1
Annoying – agreeable	6.03 (1.61)	4	6.27 (1.61)	2	5.85 (1.95)	0	5.97 (1.80)	0
Uncomfortable – comfortable	5.87 (1.67)	5	5.97 (1.68)	3	5.78 (1.87)	0	5.65 (2.02)	1
Old fashioned – modern	6.21 (1.80)	6	6.61 (2.17)	1	6.46 (1.88)	2	6.87 (1.91)	1
Unimportant – important	**8.48** (1.84)	4	**8.25** (1.74)	2	**8.28** (1.86)	0	**8.58** (1.61)	2
Bad – good	**8.44** (1.60)	3	**8.21** (1.55)	3	**8.15** (1.68)	1	**8.14** (1.73)	1
Useless – meaningful	**8.60** (1.52)	3	**8.27** (1.76)	2	**8.36** (1.55)	0	**8.60** (1.65)	1
Unattractive – attractive	**5.26** (2.18)	6	5.62 (2.24)	1	**4.91** (2.33)	3	**4.86** (2.29)	0
Stupid – fun	5.89 (2.10)	3	6.12 (2.03)	2	5.61 (2.20)	0	5.73 (2.27)	0
Placement scale^a^								
Very little – much variety in the caregiving	5.88 (2.46)	22	6.85 (2.31)	7	5.58 (2.48)	2	5.79 (2.42)	9
Very little – much contact with mentor	6.35 (2.12)	30	5.63 (2.15)	13	**4.73** (2.07)	8	**4.46** (2.04)	13
Very few – many opportunities to learn new things	6.90 (2.00)	19	6.81 (2.03)	7	5.56 (2.13)	3	5.96 (2.16)	5
My mentor will have very little – much time to evaluate	6.15 (1.84)	52	5.92 (2.00)	16	**5.16** (2.10)	11	**4.92** (2.18)	17
No – many possibilities to plan own learning activities	6.00 (1.92)	61	6.53 (2.15)	17	6.15 (2.08)	10	6.13 (2.11)	12
Profession scale^a^								
Very few – many enjoyable relationships with patients	7.68 (1.57)	14	7.54 (1.61)	5	7.64 (1.52)	1	7.77 (1.63)	3
Very little – much physically demanding work	6.85 (1.59)	14	7.09 (1.58)	5	7.06 (1.85)	2	7.20 (1.73)	2
Very little – much collaboration with colleagues	5.57 (2.42)	10	**5.42** (2.25)	4	**4.50** (2.20)	4	**4.48** (2.03)	2
Very little – much collaboration with other disciplines	6.44 (2.02)	23	7.53 (1.94)	4	6.07 (2.17)	2	6.12 (2.16)	6
Very few – many technical skills needed	7.58 (1.89)	4	6.91 (1.98)	5	5.96 (1.84)	0	6.30 (1.85)	4
Very little – a lot of freedom of action	7.16 (1.72)	18	7.98 (1.55)	5	7.75 (1.60)	3	7.84 (1.73)	5
Very little – a lot of variety in the caregiving	5.99 (2.04)	15	7.03 (1.98)	3	5.93 (2.06)	0	5.85 (2.02)	7
A poor – good occupational work environment	5.84 (1.80)	61	**5.04** (1.89)	17	**4.59** (2.03)	9	**4.15** (1.96)	8
Very little – plenty of individual responsibility	**8.20** (1.32)	8	**8.48** (1.54)	2	**8.58** (1.34)	0	**8.51** (1.20)	2
No – continual feelings of work pressure	6.87 (1.67)	21	7.16 (1.74)	6	7.29 (2.00)	2	7.40 (1.53)	4
Very few – plenty of complex patient care needs	6.22 (1.93)	25	6.58 (1.85)	5	5.85 (1.90)	1	6.02 (1.84)	5
Very few – only elderly patients	**8.81** (1.25)	3	**8.32** (1.63)	3	**8.66** (1.47)	0	**8.62** (1.23)	1
Low – high status work	**5.36** (1.81)	44	5.63 (2.00)	16	**5.23** (1.80)	6	**5.21** (1.81)	13
No – a lot of possible health improvement for the patient	6.37 (1.62)	44	6.79 (1.69)	10	6.70 (1.71)	3	6.49 (1.79)	6
Very few – many enthusiastic colleagues	6.64 (1.78)	47	7.10 (1.63)	12	6.34 (1.71)	11	6.29 (1.71)	18
Very few – much contact with family/ kin	7.70 (1.70)	13	**8.02** (1.71)	5	7.67 (1.70)	2	7.90 (1.62)	6
No – many opportunities for advancement	5.72 (2.29)	31	5.89 (2.21)	10	**5.44** (2.17)	7	**5.33** (2.23)	11

^a^The option ‘I don’t know’ (value 11) in the placement and profession scale is excluded in the calculation of the mean and defined as missing, which explains the larger/ fluctuating numbers of missing values in the placement and profession scale

At the level of the separate items, it is noticeable that the item means, specifically in the affective component scale and the profession scale, mutually differ substantially (see Table 2 in bold). In the affective component scale, the items ‘important’ (T0–3 resp. 8.48–8.58), ‘meaningful’ (8.60–8.60) and ‘good’ (8.44–8.14) have a relatively high score, while items representing a more personal attraction, such as ‘attractive’ (5.26–4.86) and ‘fun’ (5.89–5.73) score lower. The results in the profession scale show that students expect to provide care mainly to elderly patients (8.81–8.62) while carrying a lot of responsibility (8.20–8.51) in a poor occupational work environment (5.84–4.15) with little collaboration (5.57–4.48). Students also see community nursing as a low status job (5.36–5.21) with few opportunities for advancement (5.72–5.33).

The longitudinal description at item level shows that, in the affective component scale, the mean in each item after T0 hardly fluctuates over time. In the placement scale, a decline after T0 is visible, specifically in the two items ‘contact with mentor’ and ‘time to evaluate with mentor’ (mean difference 1.89 resp. 1.23, and score at T3 4.46 resp. 4.92). In the profession scale, a slight increase from T0-T3 is found in items that seem to represent two larger areas. The first is ‘contacts’, represented in the items ‘enjoyable relationships with patients’ (7.68–7.77) and ‘contact with family/kin’ (7.70–7.90). The second has a more negative connotation as it appears to relate to ‘workload’, with the items ‘physically demanding work’ (6.85–7.20), ‘work pressure’ (6.87–7.40), ‘responsibility’ (8.20–8.51) and ‘freedom of action’ (7.16–7.84). All other items decline between T0 and T3, except ‘possible health improvement’ with a very slight increase (Table 2).

### Effect of time T0-T3 on students’ perceptions of community care

To measure the effect of time on students’ perceptions of community care (SCOPE, total scale), a linear mixed model analysis (LMMs) was carried out. The type lll *F*-test for the linear trend of the relation between ‘time’ and ‘perceptions of community care’ shows a significant result at the 5% level [*F*(3, 184.308) = 19.485, *p* < .001, indicating that the relationship between the two variables varies significantly. The estimated marginal means of nursing students’ perceptions of community care (total scale) are 6.651 for T0, 6.694 for T1, 6.142 for T2 and 6.200 for T3 (Table 3).

**Table 3:** Mixed model analysis for the effect of time (T0–3) on nursing students’ perceptions of community care

	*B*	*SE B*	95% Confidence Interval
Constant	6.200	.079	6.044–6.355
T0	.451	.083	.287–.615
T1	.494	.083	.331–.657
T2	−.058	.076	−.207–.092
T3	Ref	0	–

In order to make it easier to interpret the regression coefficient *B* in the mixed model analysis, a pairwise comparison of the estimated marginal means of students’ perceptions of community care between the time points (indicating the slopes) was conducted. These values increase slightly between T0 and T1 and T2 and T3. Between T1 and T2, a significant decline is shown (Table 4).

**Table 4:** Pairwise comparison of estimated mean differences in students’ perceptions of community care at T0-T3

Time point I	Time point J	Mean difference (I – J)^a^	*p*	95% Confidence Interval
0	1	−.043	.618	−.211–.126
1	2	.552	< .001*	.372–.732
2	3	−.058	.447	−.207–.092

^a^ A negative result in mean difference indicates a positive slope

*p < .05

### Zooming in on students with a preference for community care

**Fig. 2:**
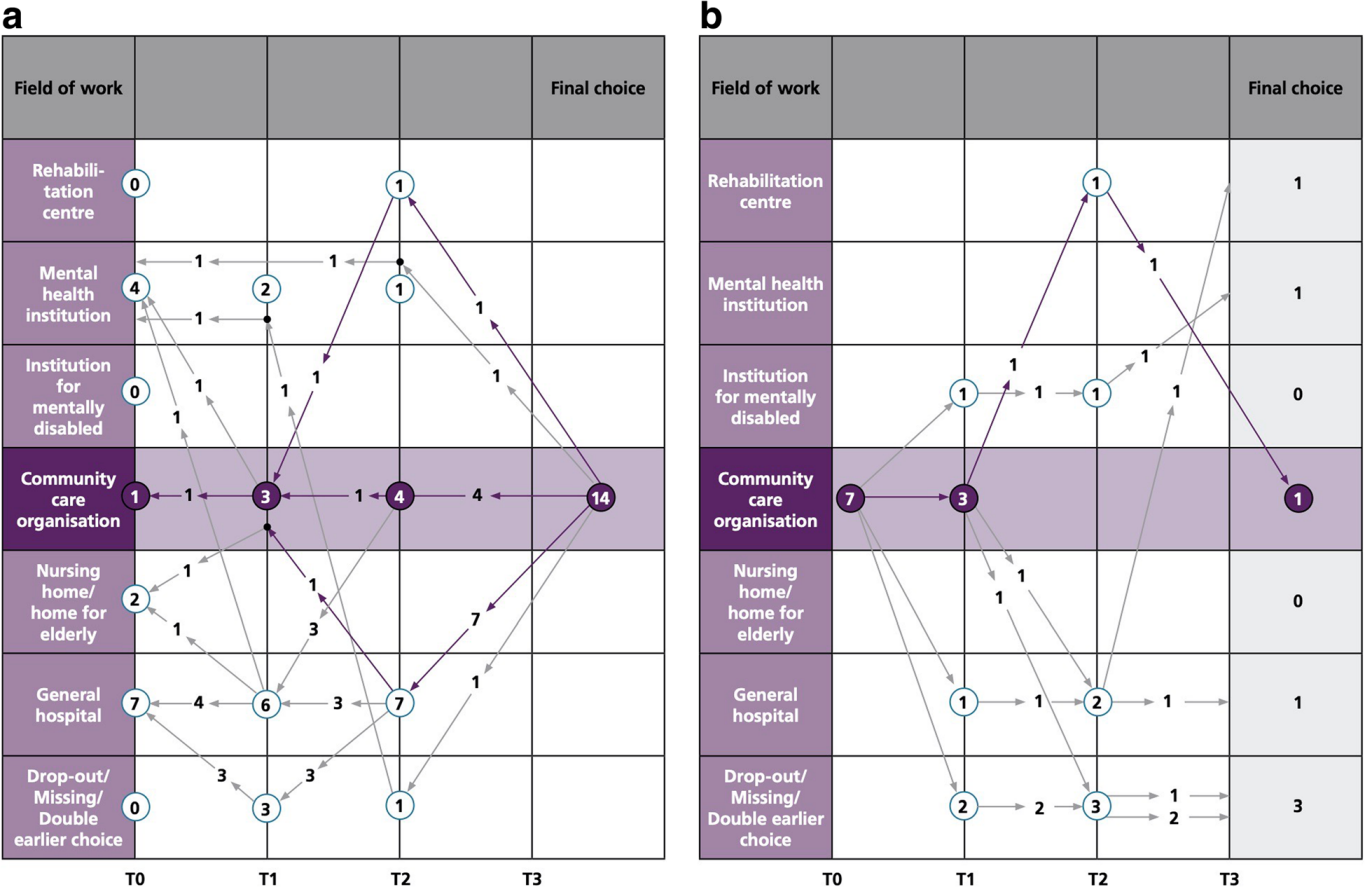
**a** Placement preferences of students choosing community care at T3 (*n* = 14), in retrospect to T0. **b** Placement preferences of students choosing community care at T0 (*n* = 7) and further to T3

The following schemes (Fig. 2a and b) represent the individual development of students’ placement preferences. The numbers in the vertical lines represent how many students prefer a specific field at each time point (T0–3). The arrows show how students move between the time points, and the numbers in the arrows how many students take this pathway. The results at individual level show that of the 14 students with a placement preference for community care at T3, only one had such preference at T0 (Fig. 2a). This is identical to the 7 students with a preference for community care at T0, as only 1 remains at T3 (Fig. 2b); the other 6 make other subsequent choices (3 students) or drop out (3 students). Apparently, although the preference for the field in the cohort generally increases from 7 to 14, there is no consistent choice for the field of community care. With regard to the perception of community care, the 14 students preferring community care at T3 have a mean in perceptions (total scale) of 7.66, compared to 6.21 in all students (Table 2).

### Placement preferences for community care at individual level

Comparing placement preferences of the 14 students preferring community care at T3 (Fig. 2a) with their factual placements at T1, 2 and 3, shows that 6 students at some point during study had a placement in community care (10 or 20 weeks). The same result is visible at other time points: of the 16 students preferring community care at T1, 2 had a placement in the field, and at T2 (*n* = 6) also 2 students. Therefore, no relation between a factual placement in community care and a preference for the field was found.

### A preference for the hospital at individual level

**Fig. 3:**
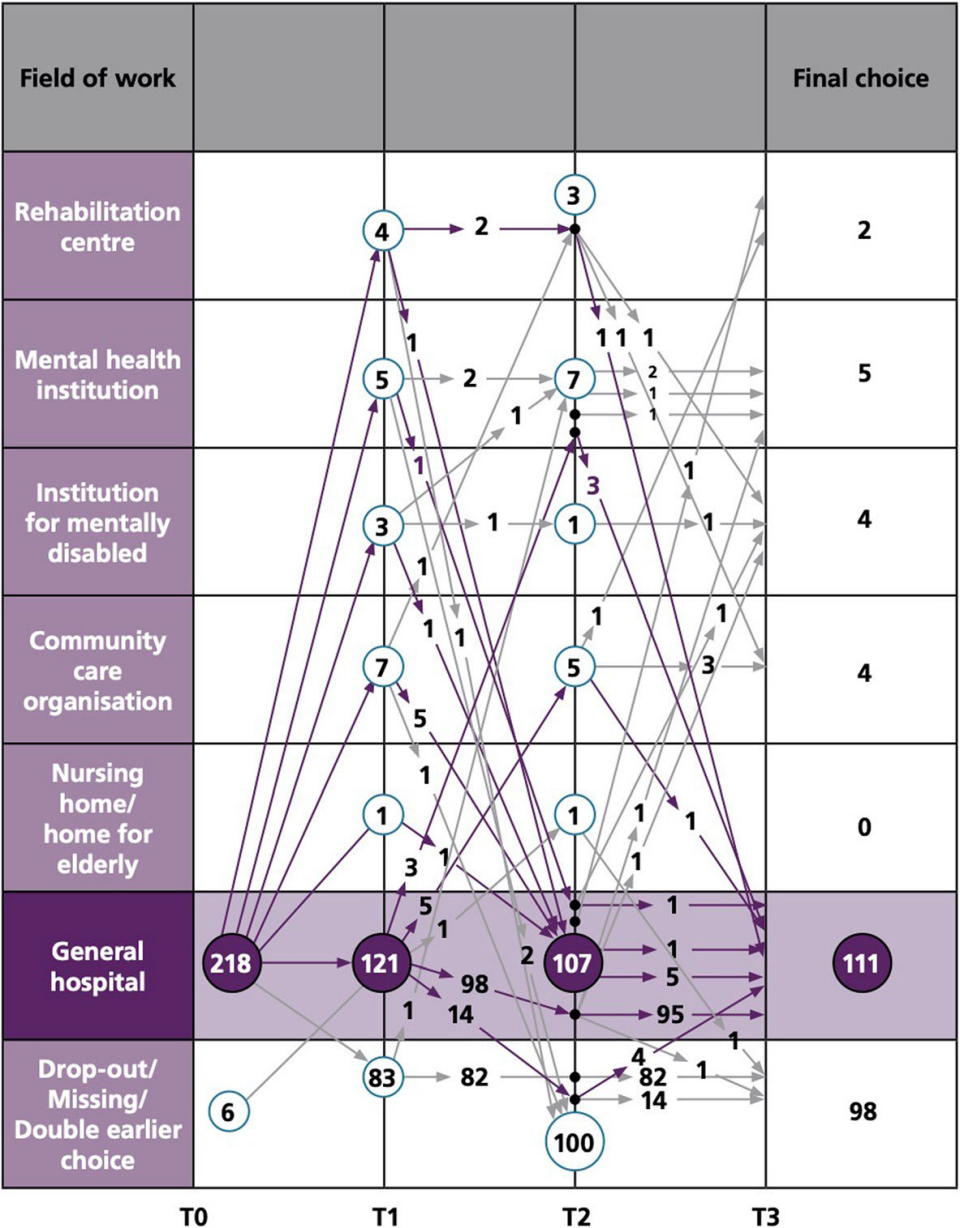
Placement preferences of students choosing the general hospital at T0 (*n* = 218)

The development of placement preferences in students choosing the general hospital at T0 (*n* = 218) provides a different pattern (Fig. 3). The number of students who drop-out is relatively large, which is, although undesirable, not uncommon in higher education in the Netherlands [41]. However, of the 121 students preferring the hospital at T1, 95 students remain consistent in their choice at all time points. Many students choosing a different area once return back to their earlier choice for the hospital at the following time point. In the period of graduation (T3), 111 students have a preference for the hospital, compared to 15 students choosing a different area.

## Discussion

The objective of this study was to investigate how baccalaureate nursing students’ perceptions towards community care and placement preferences develop during a more community care-oriented curriculum, and which curriculum elements have the potential to positively influence students’ perceptions of community care.

First, the development of students’ perceptions while progressing through the four-year curriculum (between T0 and T3) show an overall slight decrease (6.651 at T0 vs. 6.200 at T3, range 1–10), indicating that the curriculum as a whole did not have a positive effect. The higher score at T1 (6.694) could be the result of the theme week on community care (D in Fig. 1), which is a relatively intensive and ‘powerful’ intervention. However, this effect does not last. The longitudinal picture of students’ placement preferences also shows that the curriculum in period A/years 1 and 2 did not have the intended effect of students choosing the ‘paved way to community care’ in period B/years 3 and 4, indicated by the ‘dip’ at T2 in the preference for the field (Table 1). Also, in Fig. 2b, the field of community care at T2 is empty. These results correspond with existing literature on the effect of specific courses/programs on students’ perceptions of working with elderly [24–27] and in community care/primary care [23, 42], not only with regard to the positive, but also the short-term effect. The fact that most students chose for another minor program in year 3 also appeared to contribute to the significant decline between T1 and T2: education on a different theme or healthcare area might have shifted the students’ attention away from the field of community care.

Second, despite the lack of more positive perceptions in the whole cohort, the proportion of students with a preference for a placement in community care increases substantially between T0 and T3 (2.6% vs. 8.2%). This in contrast with all the other fields experiencing, to a greater or lesser extent, reduced attention. This result ties in with Pfarrwallers et al.’s conclusion that a longitudinal approach is the only effective strategy to stimulate student interest [12]. On the other hand, the results here do not provide an obvious explanation for the increased interest for community care, due to the fluctuation of students with a preference for the field during the four-year curriculum (7–16–6-14) and the fact that the 14 graduates mainly are different persons than the 7 with a community care preference at T0. However, the fact that these 14 students with a preference for community care have a higher score on perceptions than the whole cohort (7.66 vs. 6.21), indicates that placement preference and positive perceptions go ‘hand in hand’, which is in line with existing literature [7, 15].

Third, no impact of a factual placement in community care on students’ intention to work in the field is visible in this cohort. This contrasts with several studies, where students who participated in a placement were more likely to intend to work in community care [7, 10, 21, 43], and with the last placement being crucial [44]. With regard to placement experiences in this student cohort, it seems noteworthy that the items in the placement scale on ‘contact with mentor’ and ‘time to evaluate’ (Table 2) have a low score at T0, but even lower at the end (< 5). This raises doubts about the quality of students’ experiences in community care, even more so while they see clinical nurse commitment [45, 46], a qualified and motivated mentoring nurse [5, 19, 37], and a feeling of appreciation and connection with the staff [47] as important for their placement. In contrast, stress and fatigue in staff appears to be a hindrance to feeling part of a team, which negatively effects students’ valuation [48]. It is possible that the nursing students during the placement period are confronted with the consequences of labour market problems, leading to a negative vicious circle of shortages causing more shortages, when students experience a high workload and other related problems and, hence, make other career choices.

Fourth, the visualisation schemes indicate that many first-year students are undecided about their career, as their placement preferences fluctuate considerably in the subsequent years of study, a phenomenon earlier described in studies in Norway [35] and Australia [4]. Nevertheless, it should be noted that in this study, this applies to a minor extent to students with a preference for the hospital.

Finally, and in contrast with the placement preferences, students’ perceptions of community care seem to be quite stable. The data in Table 2 show that, although the items differ mutually, from a longitudinal perspective the changes per item are limited. Also, the results at T3 correspond with data collected from students graduating in 2015, 2016 and 2017 from an earlier study [29]. Difficult issues are visible hindering a positive outlook on community care, such as having much responsibility while working alone with little guidance, a low status, and few opportunities for advancement. Despite the transforming healthcare delivery environment, the status quo of the hospital being ‘the place to be’ seems difficult to modify.

These insights underline the importance of a close collaboration between educational institutions and community care organisations, as it is a growing challenge to offer good placements in a tightening labour market. Also, as students do not look forward to the high responsibility they have to bear in this more autonomous role, it may be worthwhile investing time and effort in designing a curriculum that attracts more mature students. Older students appear to have more positive perceptions of placements outside traditional settings [10, 19], and they see workplace support as less important [10]. This curriculum should, on the one hand, contain the same themes with regard to extramural caregiving, but, on the other, establish strong ties with the students, for example in a part-time baccalaureate programme for vocational trained nurses, or a dual track, combining workplace learning with learning in educational institutions. More maturity might make it easier to tackle the high responsibility that is seen as a major impediment by the younger students in the full-time programme. It could even be that more mature students see aspects as autonomy and freedom of action as desired features the hospital cannot offer them.

### Strengths and limitations

Strengths of this study are its high response rate compared to many other studies, and the holistic/ broad longitudinal approach of the curriculum-redesign. Another strength is that a longitudinal study on placement preferences with quantitative data at the individual level is uncommon. Some methodological problems limit the findings and interpretations of this study. The sample was drawn from one cohort in a single institution, and nursing curricula mutually differ, which limits the general applicability of the findings. Second, the small number of students in the ‘community care pathway’ was a limitation for some statistical procedures. Third, the repeated data collections may be considered as an intervention as well, and it is not easy to properly assess the impact on students of being ‘under scrutiny’.

### Implications for further research

Some issues with regard to students’ preferences for healthcare areas, and directly related, career choice, need further exploration. Little is known about the exact moment at which the definitive choice for an area for the future career takes place, and under what influences. These questions could be explored in a retrospective qualitative study with graduating students. Also, as the evidence on the positive effect of maturity on a preference for working outside traditional settings is limited, this topic needs further exploration.

## Conclusions

A curriculum redesign with more elements of community care stimulating a positive interest in this field resulted in two contradictory findings: it did not positively influence students’ perceptions, while the preference for a placement in this area increased substantially. Students’ placement preferences with regard to healthcare areas fluctuate considerabely while progressing through the curriculum, although a preference for the hospital appears to be more consistent. A placement in community care did not have an impact on students’ choice for the field. A strong intervention in the form of an intensive theoretical programme was temporarily successful in stimulating interest for community care, but this effect faded. Apparently, more is needed to attract this relatively young type of student for the field of community care. In addition, other curricula, for example dual learning pathways for more mature students and/or nurses with a vocational training may be an alternative contribution to solving the labour market problems in community care.

## Figures and Tables

**Fig. 4:**
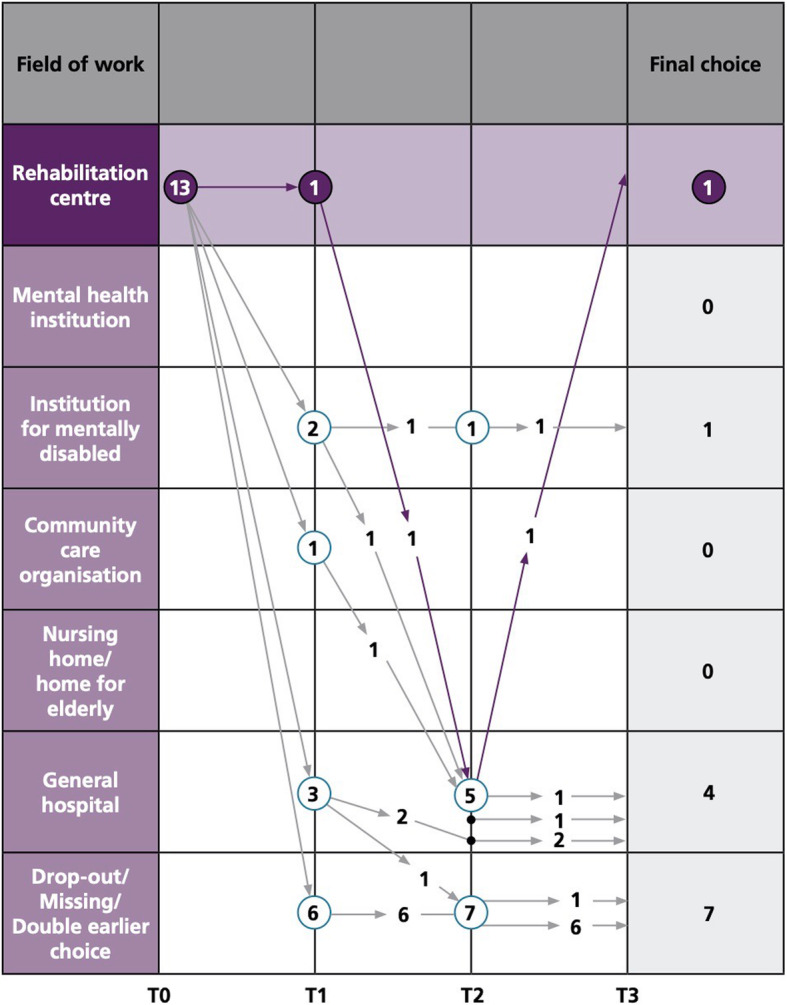
Development of placement preferences of students with a preference for rehabilitation centre at T0

**Fig. 5:**
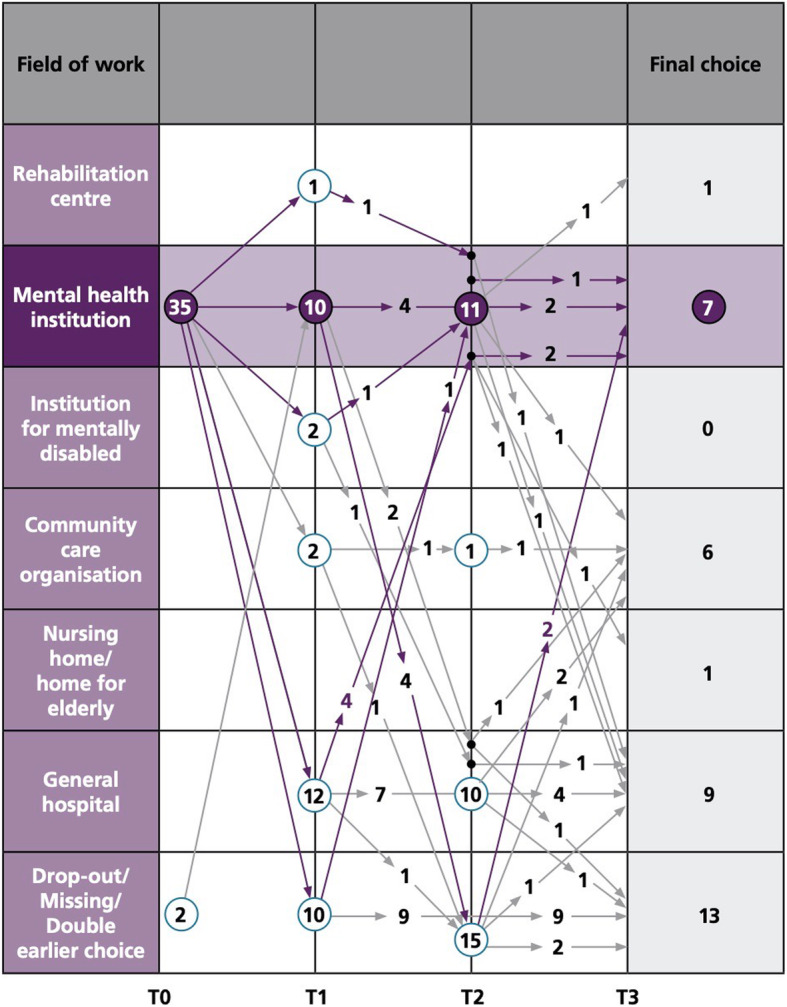
Development of placement preferences of students with a preference for mental health institution at T0

**Fig. 6:**
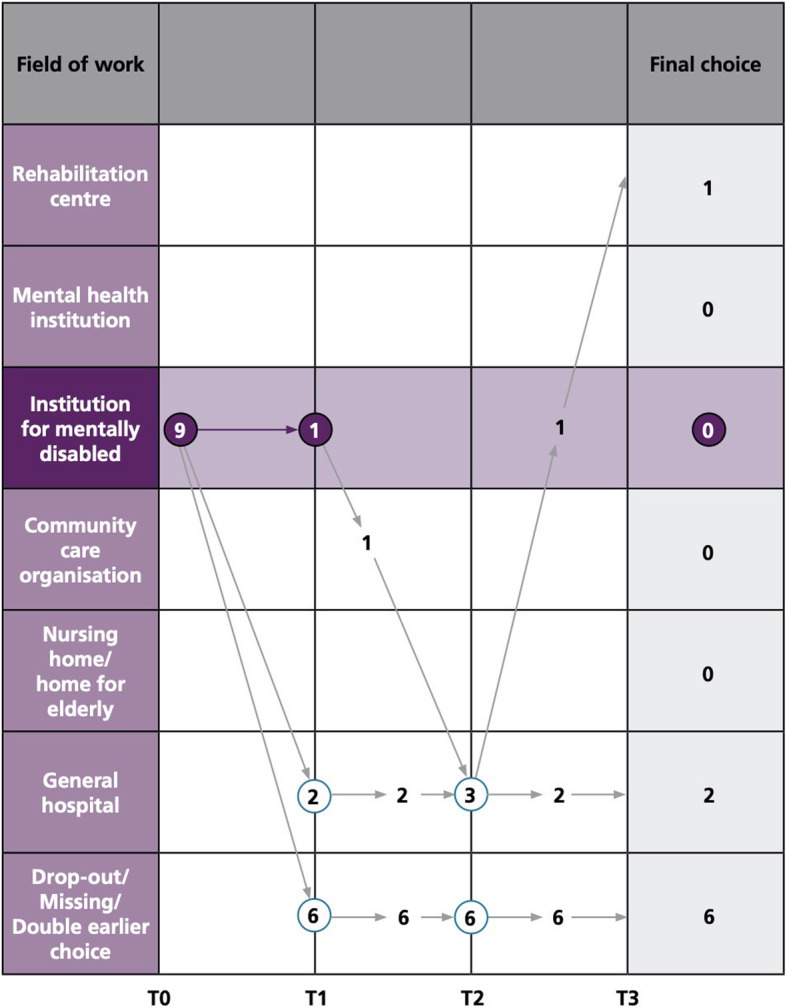
Development of placement preferences of students with a preference for institution for mentally disabled at T0

**Fig. 7:**
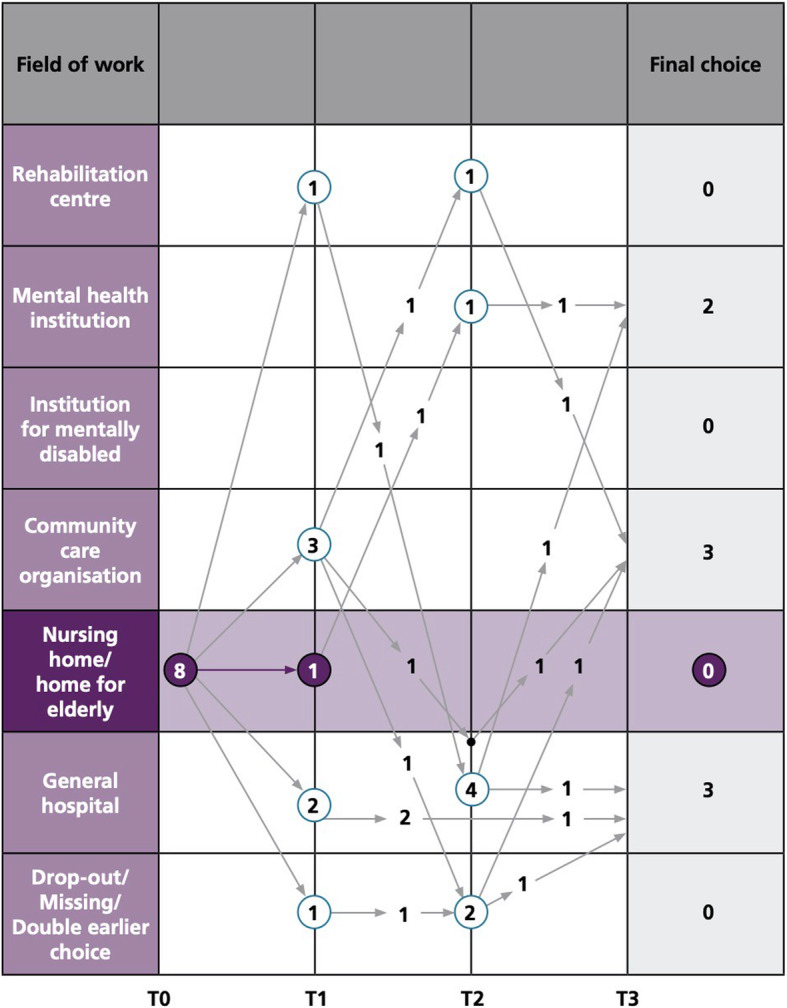
Development of placement preferences of students with a preference for nursing home/ elderly home at T0

## Data Availability

The dataset supporting the conclusions of this article is available in the Figshare repository: 10.21943/auas.8152667.v1. Please contact opensciencesupport@hva.nl if you want to request the data from this study.

## Appendix

APPENDIX 1 - DEVELOPMENT OF PLACEMENT PREFERENCES T0-T3: OTHER FOUR HEALTHCARE AREAS (Figs. 4, 5, 6, 7)

## Abbreviations

DAGMAR: Defining advertising goals for measured advertising results
EC: European credits
SCOPE: Scale on community care perceptions
